# Host age and *Plasmodium falciparum* multiclonality are associated with gametocyte prevalence: a 1-year prospective cohort study

**DOI:** 10.1186/s12936-017-2123-2

**Published:** 2017-11-21

**Authors:** Yaw Adomako-Ankomah, Matthew S. Chenoweth, Aaron M. Tocker, Saibou Doumbia, Drissa Konate, Mory Doumbouya, Abdoul S. Keita, Jennifer M. Anderson, Rick M. Fairhurst, Mahamadou Diakite, Kazutoyo Miura, Carole A. Long

**Affiliations:** 1 Laboratory of Malaria and Vector Research, National Institute of Allergy and Infectious Diseases, National Institutes of Health, Rockville, MD USA; 2 Malaria Research and Training Center, Faculty of Medicine, Pharmacy, and Odontostomatology, University of Sciences, Techniques, and Technologies of Bamako, Bamako, Mali

**Keywords:** *Plasmodium falciparum*, Malaria, Gametocytes, Longitudinal, Multiclonality, Mali

## Abstract

**Background:**

Since *Plasmodium falciparum* transmission relies exclusively on sexual-stage parasites, several malaria control strategies aim to disrupt this step of the life cycle. Thus, a better understanding of which individuals constitute the primary gametocyte reservoir within an endemic population, and the temporal dynamics of gametocyte carriage, especially in seasonal transmission settings, will not only support the effective implementation of current transmission control programmes, but also inform the design of more targeted strategies.

**Methods:**

A 1-year prospective cohort study was initiated in June 2013 with the goal of assessing the longitudinal dynamics of *P. falciparum* gametocyte carriage in a village in Mali with intense seasonal malaria transmission. A cohort of 500 individuals aged 1–65 years was recruited for this study. Gametocyte prevalence was measured monthly using Pfs25-specific RT-PCR, and analysed for the effects of host age and gender, seasonality, and multiclonality of *P. falciparum* infection over 1 year.

**Results:**

Most *P. falciparum* infections (51–89%) in this population were accompanied by gametocytaemia throughout the 1-year period. Gametocyte prevalence among *P. falciparum*-positive individuals (proportion of gametocyte positive infections) was associated with age (p = 0.003) but not with seasonality (wet *vs.* dry) or gender. The proportion of gametocyte positive infections were similarly high in children aged 1–17 years (74–82% on median among 5 age groups), while older individuals had relatively lower proportion, and those aged > 35 years (median of 43%) had significantly lower than those aged 1–17 years (p < 0.05). *Plasmodium falciparum*-positive individuals with gametocytaemia were found to have significantly higher *P. falciparum* multiclonality than those without gametocytaemia (p < 0.033 in two different analyses).

**Conclusions:**

Taken together, these results suggest that a substantial proportion of Pf-positive individuals carries gametocytes throughout the year, and that age is a significant determinant of gametocyte prevalence among these *P. falciparum*-positive individuals. Furthermore, the presence of multiple *P. falciparum* genotypes in an infection, a common feature of *P. falciparum* infections in high transmission areas, is associated with gametocyte prevalence.

**Electronic supplementary material:**

The online version of this article (10.1186/s12936-017-2123-2) contains supplementary material, which is available to authorized users.

## Background

Although malaria incidence and mortality rates have steadily declined from 2000 to 2015 (62% decline in mortality rate; 41% decline in incidence rate; [1]), malaria remains a major threat to global public health, especially in Africa where > 92% of malaria-related deaths occurred in 2015 [1]. *Plasmodium falciparum,* the most lethal malaria parasite causing 99% of malaria-related deaths in 2015, is the predominant malaria parasite in Africa. Human-to-mosquito transmission of *P. falciparum* relies exclusively on the gametocyte stage of parasite development, and this step in the transmission cycle has been targeted by several malaria control strategies. A better understanding of which individuals constitute the primary gametocyte reservoir within an endemic population, and the temporal dynamics of gametocyte carriage, especially in seasonal transmission settings, is needed to support the effective implementation of existing strategies, and to further guide the design of more effective transmission control programmes.

In hyperendemic areas, where repeated exposure to *P. falciparum* infections is commonplace, individuals develop non-sterile immunity to clinical malaria over time [2]. The consequence of this partial immunity is that many people tend to carry untreated asymptomatic infections for up to several months [3–5], with varying contributions to the gametocyte reservoir. Gametocyte prevalence is not homogeneous within a population, and factors such as host age and asexual-stage parasitaemia [6–8] have been associated with gametocyte carriage in natural *P. falciparum* infections. A recent systematic review has shown that younger age, lower asexual parasite density, lower haemoglobin concentration, and absence of fever were risks for gametocyte positivity in uncomplicated malaria patients before artemisinin-based combination therapy [9]. The existing gametocyte prevalence data derive mostly from cross-sectional studies and rely heavily on microscopic analysis of blood smears, a method well documented for its limited sensitivity. Infections with submicroscopic gametocytaemia constitute a significant component of the transmission reservoir in many endemic areas [10–12]. Nevertheless, there is limited information on longitudinal gametocyte carriage measured by sensitive molecular methods, particularly in areas with seasonal variation in transmission intensity [13].

To gain a better understanding of the year-round dynamics of *P. falciparum* infections (including total parasite and gametocyte carriage) in a seasonal transmission area, a 1-year prospective cohort study was conducted in Kenieroba, Mali, from June 2013 to May 2014 [14]. Gametocyte prevalence was measured at monthly intervals using gametocyte-specific RT-PCR, and then tested for association with several host and parasite factors.

## Methods

### Ethical statement

Ethical clearance for this study was provided by the Institutional Review Board of the National Institute of Allergy and Infectious Diseases and the Ethics Committee of the Faculty of Medicine, Pharmacy, and Odontostomatology, University of Bamako. The study is registered with Clinicaltrials.gov (Identifier No. NCT01829737). Written informed consent was obtained from all participants aged ≥ 18 years, or the parents or guardians of individuals aged < 18 years.

### Study site and population

In June 2013, a prospective cohort study was initiated in Kenieroba, Mali, to assess the population-level dynamics of asexual and sexual parasite carriage over 1 year. Malaria transmission in this area is seasonal, with most cases occurring during the June-December rainy season [14, 15]. The remainder of the year is mostly dry, with relatively few malaria cases reported. A cohort of 500 individuals aged 1–65 years, selected to have a similar age distribution as that of the entire village population, was recruited for the study. Enrollment was based on age and residency within Kenieroba for the 1-year study period. The average retention rate for the study period was 84%. The demographic characteristics of the cohort, and information on clinical malaria and total parasite (asexual and sexual parasites) prevalence during the 1-year study period have been previously reported [14].

### Sample collection and malaria case detection

Finger-prick blood samples were collected as dried blood spots (DBS) on Whatman 3MM filter paper and also as whole blood in RNAprotect^®^ Cell Reagent (Qiagen) for nucleic acid stabilization. Filter paper samples were stored under dry conditions at room temperature, and used for total parasite detection [14]. RNAprotect^®^ samples were stored at − 80 °C until nucleic acid extraction and molecular analyses were performed. For participants who reported malaria symptoms (i.e., history of fever, headache, body ache, and/or malaise) at any time during the study, thin and thick blood smears were prepared, Giemsa-stained, and read by experienced microscopists for parasitological diagnosis. Confirmed cases were referred to the Kenieroba Health Centre for standard-of-care anti-malarial treatment after samples collection. It should be noted that microscopic detection of parasites was only performed on samples collected from such symptomatic individuals for parasitological diagnosis of malaria cases, and not for general detection of *P. falciparum* infection in asymptomatic individuals in this study.

### Molecular detection of *Plasmodium falciparum* infection and gametocyte carriage

DBS samples were screened for *P. falciparum* infection by species-specific nested-PCR as previously described [14]. Using whole blood samples collected in RNAprotect^®^ Cell Reagent, *P. falciparum* gametocyte prevalence was measured by RT-PCR targeting expression of the female gametocyte specific *Pfs25* gene. Total RNA extraction was performed using the RNeasy Plus 96 Kit (Qiagen), with an additional on-column DNase I treatment to eliminate any remaining contaminating genomic DNA (gDNA). RNA was eluted in 100 µL of RNase-free H_2_0 and stored at − 80 °C until molecular assays were performed. Prior to the RT-PCR assay for gametocyte detection, all extracted RNA samples were tested for gDNA contamination with a 40-cycle Pfs25-targeted PCR, which was analysed with the LabChip GX and the HT-DNA 5 K LabChip capillary electrophoresis setup in accordance with the manufacturer’s protocol (Caliper Life Sciences). After confirming absence of gDNA, RNA samples were used as templates in a 1-step Pfs25-targeted RT-PCR assay for gametocyte detection. Primers and probe used in this assay were previously described by Wampfler et al. [16]. The RT-PCR assays were performed on CFX Connect™ Real-Time PCR Detection System (Bio-Rad), with the One Step PrimeScript™ RT-PCR Kit (Clontech). Each reaction included 7.5 µL of reaction buffer, 0.3 µL each of Ex Taq and RT enzymes, 400 nmols of each primer, 200 nmols of probe, and 2 µL of extracted RNA for a total reaction volume of 15 µL. The following thermocycler protocol was used: 1 cycle of 42 °C for 5 min and 95 °C for 10 s, 45 cycles of 95 °C for 5 s and 58 °C for 30 s. Assays were performed in duplicates for each sample. The threshold for positivity was set at 40 cycles based on testing with quadruplicate serial dilutions of a plasmid standard carrying one copy of the Pfs25 target sequence [16]: the lower detection limit of this assay occurred at a one plasmid copy per µL concentration with an average threshold cycle (Ct) of 40 cycles (StDev = 0.19). This limit of detection is equivalent to 0.05 gametocytes/µL [16]. Therefore, samples with one or both replicates at Ct of < 40 cycles were considered positive. No-template negative controls were included in each run.

### Identification of multiclonality by SNP DNA barcoding

Multiclonality was assessed among Pf-positive samples at four time-points (June, November, February, and April), which were selected to reflect periods of varying transmission intensities during the year. Multiclonality was measured using a SNP DNA barcode assay as previously described [14, 17]. *Plasmodium falciparum* multiclonality was reported for each sample in two ways, as described elsewhere [14]. In brief, polymorphic proportion (PmP) was defined as the proportion (%) of polymorphic reactions among the total number of successful reactions (reactions that yielded monomorphic or polymorphic nucleotide calls) from the 24 reactions that comprise the 24-SNP barcode assay, and complexity of infection (COI) was the estimated number of unique *P. falciparum* genotypes in each infection calculated by the COIL program [14].

### Data analysis

Cross-sectional Pf gametocyte prevalence was calculated at each time-point in two ways; (1) the number of gametocyte-positive blood samples out of the total number of blood samples collected at the respective time-point*, termed “population gametocyte prevalence” in this manuscript,* and (2) the number of gametocyte-positive samples out of *P. falciparum*-positive (by nPCR) blood samples (called “proportion of gametocyte positive infections”). The latter measure was used for all statistical analysis. The proportion of gametocyte positive infections between wet (June to December) and dry (January to May) season time-points were compared by Mann–Whitney test. To analyse the effect of age on proportion of gametocyte positive infections, the cohort was categorized into seven age groups such that each group had a similar number of individuals (60–91 in each group). Proportion of gametocyte positive infections in each age group was then determined at each of the 12 time-points (one data point per month). Age-effect (12 proportion data for each age group) was analysed by One-way ANOVA test followed by Tukey’s multiple comparison test. Similarly, the proportion of gametocyte positive infections in each gender was calculated for each of the 12 time-points, and the gender-effect (n = 12 for each gender) was evaluated using Mann–Whitney test. Multiclonality was compared between gametocyte-positive and -negative samples using Mann–Whitney test for PmP, and Chi square test for COI.

## Results

### Clinical malaria incidence and population *Plasmodium falciparum* gametocyte prevalence

**Fig. 1:**
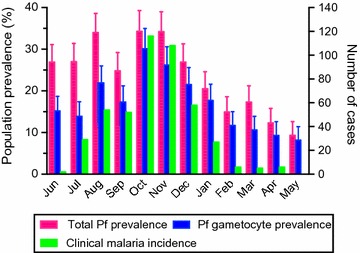
Population total *Plasmodium falciparum* prevalence, *P. falciparum* gametocyte prevalence and clinical malaria incidence. Total *P. falciparum* prevalence (include both asexual and sexual parasites) and *P. falciparum* gametocyte prevalence in the entire cohort at each month for 1 year are shown with the 95% confidence intervals (left y-axis). Total *P. falciparum* positivity was measured by species-specific nested PCR, and, *P. falciparum* gametocyte positivity by Pfs25 mRNA RT-PCR. In addition, the number of malaria cases over each 1-month period preceding sample collection is shown (right y-axis)

During the 1-year study period, a total of 469 clinical malaria cases were recorded among 232 individuals; viz. 46.4% (232 out of 500) of all study participants experienced at least one clinical malaria episode during the year [14]. The highest monthly incidence of 116 malaria episodes was measured in October, and the lowest incidence of no malaria episodes in May 2014 (Fig. 1). Nearly all *Plasmodium* infections were caused by *P. falciparum* (*Plasmodium malariae* was the only other species detected, at ≤ 5% prevalence throughout the year) [14]; therefore, only *P. falciparum* parasites (total *P. falciparum* positivity and *P. falciparum* gametocyte positivity) were evaluated in this study, irrespective of potential, but rare infections by other *Plasmodium* spp.

Population *P. falciparum* prevalence (by nested PCR) was previously determined at 2-week intervals within this cohort over the 1-year study period [14]. Here, gametocyte positivity was assessed at one of two time-points for each month by Pfs25-targeted RT-PCR among *P. falciparum*-positive samples (i.e., samples showing parasitaemia as detected by nested-PCR, regardless of their clinical status). Cross-sectional *P. falciparum* gametocyte prevalence in the entire cohort (population gametocyte prevalence) showed a seasonal profile, ranging from a minimum of 8.3% in May 2014 to a maximum of 30.1% in October 2013, similar to the seasonal effect seen in population total *P. falciparum* prevalence (Fig. 1).

### Gametocyte positivity among *P. falciparum* carriers

**Fig. 2:**
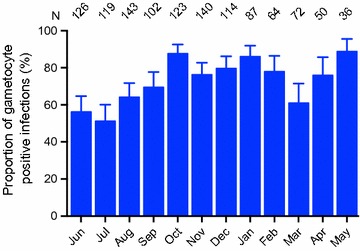
Monthly proportion of gametocyte positive infections over 1 year. Proportion of gametocyte positive infections among all *P. falciparum* positive individuals were assessed once per month, regardless of their treatment status. The error bars indicate the 95% confidence intervals. Number of *P. falciparum*-positive individuals screened for gametocyte-positivity at each month is shown at the top

Figure 2 shows gametocyte prevalence among all *P. falciparum*-positive individuals (proportion of gametocyte positive infections) at one-month intervals over 1 year. The proportion of gametocyte positive infections ranged from 51.3% (61/119, July 2013) to 88.9% (32/36, May 2014), with no significant effect of seasonality on proportion (wet *vs.* dry season, p = 0.343, Mann–Whitney test). A previous assessment of the impact of several host factors, including age, gender, and red blood cell polymorphisms (i.e., ABO/Rh types, haemoglobin phenotype, and G6PD deficiency and α-thalassaemia genotypes) identified significant age and gender effects on persistent total *P. falciparum* positivity, while all other factors tested showed no significant association [14]. Since more than half of *P. falciparum*-positive individuals were identified as gametocyte-positive throughout the year, when cross-sectional population gametocyte prevalence was analysed, similar age and gender effects were observed on gametocyte positivity as was observed on total *P. falciparum*-positivity ([14], Additional file 1). Therefore, subsequent analyses were performed using proportion of gametocyte positive infections, not the population gametocyte prevalence.

### Effects of age and gender on proportion of gametocyte positive infections

**Fig. 3:**
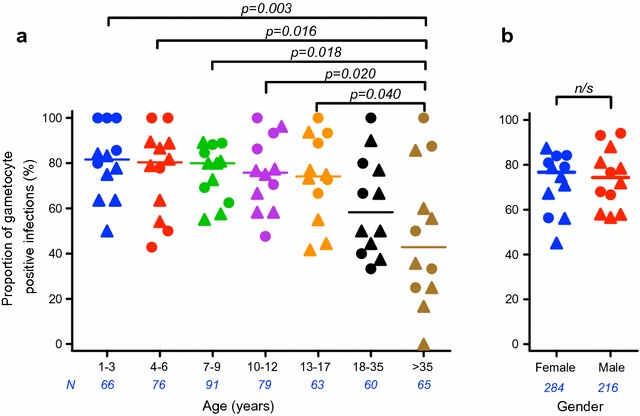
Proportion of gametocyte positive infections stratified by age (**a**) and gender (**b**). Each data-point represents the proportion among the respective age group at one of 12 time-points over the 1-year period. Number of individuals (*N*) in each age and gender group is shown in blue. Triangles represent wet season time-points (June–December) and circles represent dry season time-points (January–May). **a** There was a significant age-effect on proportion of gametocyte positive infections (p = 0.003, One-way ANOVA). Groups of children aged 1–17 years have similarly high proportions. The > 35 age-group has significantly lower proportion than age-groups ≤ 17 years (each of all age groups was compared to all other groups by Tukey’s multiple comparison tests, and only significant p-values are shown). **b** There was no significant difference in gametocyte proportion between males and females (p = 0.755, Mann–Whitney test)

To assess age effect, the cohort was categorized into seven age groups and the proportion was measured for each age group at each of the 12 months during the year (Fig. 3a). Proportion of gametocyte positive infections was similarly high (74–82%, median of 12 time-points in each age group) in the five age groups ≤ 17 years. Older individuals had relatively lower proportions (medians of 58 and 43% in 18–35 and > 35 age groups, respectively), and the > 35 age group showed significantly lower proportion than age groups ≤ 17 years (p = 0.003, One-way ANOVA; p < 0.05, Tukey’s multiple comparison test). Similarly, the proportion of gametocyte positive infections in each gender group was determined at 12 time-points (Fig. 3b), and there was no significant difference between males and females (p = 0.755, Mann–Whitney test). In both analyses, there was no appreciable effect of seasonality on proportion (Fig. 3). Furthermore, none of the RBC polymorphisms listed above were associated with gametocyte positive rate (number of gametocyte positive samples among all *P. falciparum* positive samples tested throughout a year in each individual) in a multiple linear regression analysis after adjusting for age and gender (Additional file 2). Out of the 1176 *P. falciparum*-positive samples, 57 RNA samples were collected from participants with symptomatic malaria at the time of sampling. Of those, 54 (94%) samples were identified as gametocyte positive. Hence, the chance of gametocyte positivity in symptomatic individuals was significantly higher than that in asymptomatic individuals (786/1119, 70%; p < 0.0001 by a Fisher’s exact test).

### Relationship between *P. falciparum* multiclonality and gametocyte carriage

**Fig. 4:**
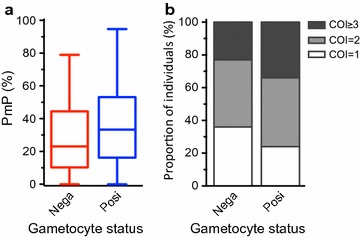
Comparison of multiclonality between gametocyte-negative (Nega) and –positive (Posi) individuals among *P. falciparum*-positive individuals. **a** Polymorphic proportion (PmP, proportion of polymorphic reactions among the total number of successful reactions) and **b** complexity of infection (COI, COIL-estimated number of unique *P. falciparum* genotypes in each infection) among *P. falciparum*-positive individuals at four time-points (Jun, n = 119; Nov, n = 110; Feb, n = 51; and Apr, n = 41) were pooled and stratified by gametocyte status. Gametocyte positive individuals have significantly higher PmP (p = 0.009, Mann–Whitney test) and COI (p = 0.033, Chi square test) than gametocyte-negative individuals

It was previously observed that there was a significant positive correlation between persistent *P. falciparum* positivity over 1 year and multiclonality at multiple time-points throughout the year [14]. Additionally, multiclonality, as measured by either PmP or COI, remained stable between wet and dry season time-points. Here, the relationship between multiclonality of *P. falciparum* infections and gametocyte positivity was investigated. Both PmP and COI were compared between gametocyte-positive and gametocyte-negative infections at 2 wet season and 2 dry season time-points. There was a general trend of higher PmP and COI in gametocyte-positive compared to gametocyte-negative infections throughout the year, although these differences were not statistically significant at individual time-points except in November (Additional file 3). When data from all four time-points were combined, PmP and COI were significantly higher in gametocyte-positive than gametocyte-negative infections (Fig. 4a, p = 0.009, Mann–Whitney test; Fig. 4b, p = 0.033, Chi square test).

## Discussion

In this study, a highly sensitive molecular method was used to measure changes in gametocyte positivity over a 1-year period in an area with intense, seasonal malaria transmission in Mali. Subsequently, the effects of age and gender on the proportion of gametocyte positive infections, as well as the relationship between gametocyte positivity and multiclonality of *P. falciparum* infections were analysed. Most *P. falciparum* infections in this population over 1 year were found to be concurrently gametocytaemic (51–89% of Pf-positive individuals). There was no effect of seasonality or gender on proportion of gametocyte positive infections. When age-effect was assessed, the likelihood of carrying gametocytes was similarly high in groups of children aged ≤ 17 years. However, adults aged > 35 years had a significantly lower proportion of gametocyte infections than most groups of children aged ≤ 17 years, while adults aged 18–35 years showed an intermediate proportion. Interestingly, this study also found that *P. falciparum* infections with gametocytaemia had relatively higher multiclonality than those without gametocytaemia.

The current report is an expansion of a year-long cohort study conducted to investigate the longitudinal dynamics of *P. falciparum* carriage in an area where malaria transmission is intense and seasonal [14]. This included an assessment of both asexual parasites and the transmissible gametocyte stage. In a previous report, it was observed that about 38% of this population carried *P. falciparum* infections at peak transmission and that age and gender were significant determinants of Pf positivity throughout the year. Furthermore, persistent parasite carriage was significantly associated with reduced risk of developing clinical malaria over the 1-year study period [14]. In the current study, it was found that most *P. falciparum*-positive individuals in this population were also gametocyte-positive. Therefore, it was not surprising that the cross-sectional population gametocyte prevalence showed a generally similar profile over the 1-year study period as population total *P. falciparum* prevalence [14]. This observation is consistent with findings showing gametocyte prevalence to broadly follow asexual parasite prevalence in other transmission settings [9, 18–20], although this pattern of association may be affected by other factors including host age and transmission intensity [13, 21].

Interestingly, while the total number of gametocyte carriers was evidently higher in the wet season (Fig. 1), the proportion of concurrent gametocyte carriage among *P. falciparum* infections did not differ significantly between the wet and dry seasons (Fig. 2). Several studies have examined the effects of seasonality on population-level gametocyte prevalence, and in some instances, they found seasonal changes in gametocyte prevalence as a function of rainfall and changes in asexual parasite density [13, 18, 22]. This is consistent with population gametocyte prevalence observed in this study. However, it is unclear whether proportion of gametocyte positive infections is affected by the same determinants, such as rainfall and asexual parasite density, which affect population gametocyte prevalence. In a report from Southeast Asia, gametocyte prevalence among *P. falciparum*-positive individuals was found to be significantly higher in the dry season than in the wet season, and negatively correlated with rainfall [23]. Results from the present study indicate little to no effect of seasonality on proportion of gametocyte positive infections in this area of high transmission intensity. Possible explanations for this disparity include the high transmission intensity, and the use of a sensitive molecular method to detect low-density infections in this study (the Southeast Asia study used microscopic detection [23]). Overall, the high level of gametocyte prevalence among *P. falciparum*-positive individuals in this region (Fig. 2) is in line with a growing body of evidence obtained through molecular monitoring [9, 24–26], which supports the premise that a much greater proportion of *P. falciparum* infections produces gametocytes and potentially contributes to the infectious reservoir than initially deduced from studies that relied largely on microscopy for gametocyte detection [27, 28]. These findings suggest the need to consider a larger proportion of the population for targeted transmission control programmes, such as mass drug administration, to significantly impact malaria transmission.

Age was found to be a significant determinant of proportion of gametocyte positive infections in this area. However, similarly high gametocyte proportion among *P. falciparum* positive individuals was observed in children aged 1–17 years. Children have commonly been associated with higher risk of gametocyte carriage than adults [7, 8]. In some instances, peak gametocyte prevalence was measured in school-age children (5–15 years), with significantly lower prevalence in individuals outside this age range [20, 28]. In this study population, while population total parasite prevalence peaked in children aged 9–16 years (both younger and older groups showed lower prevalence) [14], the chance of carrying gametocytes once infected was similarly high in all individuals up to age 17 years, but relatively lower in adults aged ≥ 18 years. Children aged 1–17 years constitute 66% of the entire village population (village-wide census conducted in May 2012), and therefore represent a dense subpopulation of gametocyte carriers. Further studies, particularly assessing gametocyte density and mosquito infectivity measurements in each age group, will be required to determine the importance of younger age group on the actual transmission in the field.

The occurrence of multiple distinct parasite clones is a common feature of *P. falciparum* infection. Multiclonality has effects on various aspects of *P. falciparum* infection, from outcome of infection [29, 30] to transmission to mosquitoes [31, 32]. With respect to gametocyte carriage, multiclonal *P. falciparum* infections have been reported to last longer and have increased likelihood to produce gametocytes relative to single-clone infections [3], although another study found no association between genetic diversity and gametocyte prevalence [33]. In this cohort, multiclonality was higher among gametocyte-positive relative to gametocyte-negative individuals over the course of 1 year.

There are a few limitations to this study. First, the volumes of blood samples collected in this study were not standardized, making it impossible to measure total parasite and gametocyte densities. With this limitation in mind, it should be noted that several associations found in this study may be explained by asexual parasite or gametocyte densities. For example, children might be more likely to carry infections at higher asexual parasite densities, and consequently higher gametocyte positivity and also higher COI. Second, the study had limited power to evaluate the relationship between gametocyte carriage and RBC polymorphisms, such as ABO/Rh types, haemoglobin phenotype, and G6PD deficiency and α-thalassaemia genotypes. Third, the 1-year study design places a limit on the study to assess these interactions beyond a single transmission year. It may be necessary to re-evaluate the effects of various risk factors of gametocyte prevalence as transmission dynamics change over time. Fourth, gametocyte positivity was analysed only for *P. falciparum*-positive individuals (detected by nested PCR) at the time of sample collection. However, Pfs25 transcript abundance can exceed DNA copy number, therefore, this study could miss some gametocyte positive cases (with lower gametocyte density) in “*P. falciparum*-negative” samples. Lastly, direct skin feed and/or direct membrane feeding assay was not performed in this study. Therefore, gametocyte positivity may not necessarily directly link with infectivity in the field.

## Conclusions

Results from this study indicate that in this area of intense, seasonal malaria transmission, there is a high prevalence of gametocyte carriage among *P. falciparum* infections, and that individuals aged ≤ 17 years who have *P. falciparum* infections all have high likelihoods of also carrying gametocytes. These findings will help guide the design of transmission studies and intervention strategies that target the gametocyte reservoir in this and perhaps other areas with similar human population structure and *P. falciparum* transmission dynamics. Furthermore, high multiclonality is associated with high gametocyte positivity, suggesting that intra-species competition in *P. falciparum* infection favours gametocyte development in this population.

## Additional files

**Additional file 1.** Age and gender effects on cross-sectional population gametocyte prevalence among the entire cohort during the wet and dry seasons. Gametocyte positivity was measured by RT-PCR once a month for 1 year. Data from November (A) and April (B) are presented as representative time-points for wet and dry seasons, respectively. Black bars and gray bars represent the proportion of gametocyte-positives and –negatives, respectively, within each category. Similar age and gender patterns were consistently observed at all other time-points throughout the year.

**Additional file 2.** Effect of RBC polymorphisms on gametocyte positive rate.

**Additional file 3.** Comparison of multiclonality between gametocyte-negative and -positive in Pf-positive individuals at four time-points. (A) Polymorphic proportion (PmP, proportion of polymorphic reactions among the total number of successful reactions) and (B) complexity of infection (COI, COIL-estimated number of unique Pf genotypes in each infection) among Pf-positive individuals at June (n = 119), November (n = 110), February (n = 51), and April (n = 42) time-points. For PmP, red boxes represent gametocyte-negative (Nega) and blue boxes represent gametocyte-positive (Posi) individuals. PmP and COI were compared between gametocyte carriers and non-carriers using Mann–Whitney and Chi square tests, respectively. Only the November time-point showed a significant difference in PmP (*p* = *0.029*) or COI (p = 0.025) based on gametocyte status.

## Abbreviations

COI: complexity of infection
gDNA: genomic DNA
Ct: threshold cycle
Pf: *Plasmodium falciparum*
PmP: polymorphic proportion
