# Supplementary figures and images for: Host age and Plasmodium falciparum multiclonality are associated with gametocyte prevalence: a 1-year prospective cohort study
Source: Malar J. 2017 Nov 21;16:473. doi: 10.1186/s12936-017-2123-2 (PMC5696713; doi:10.1186/s12936-017-2123-2)

**A.**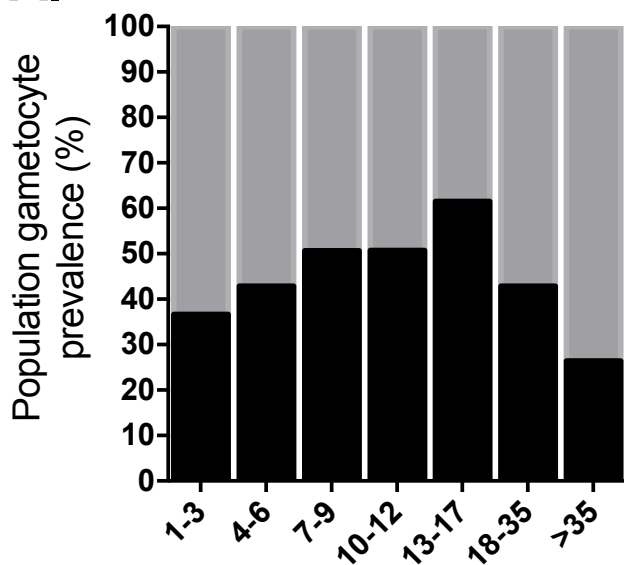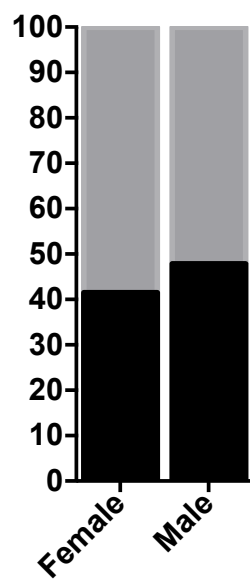**B.**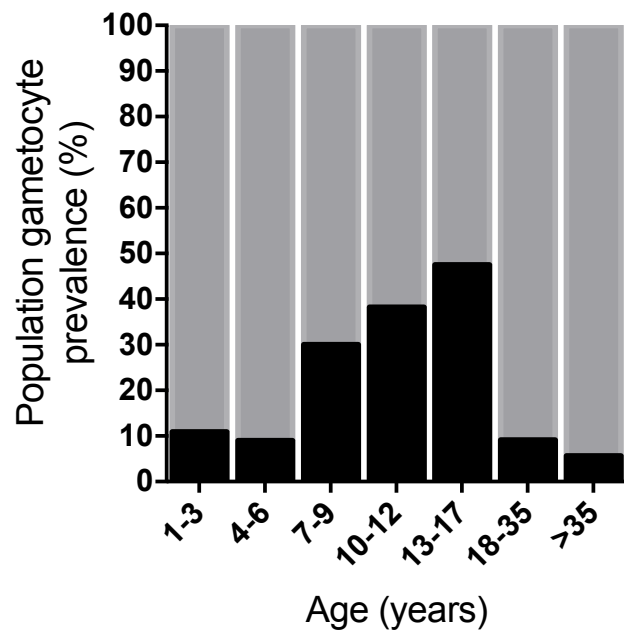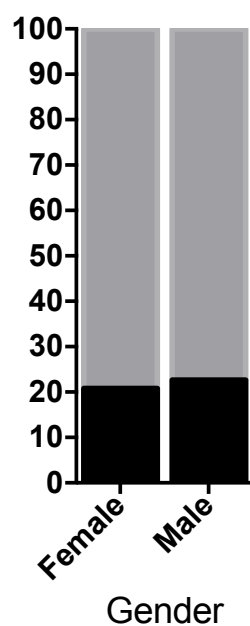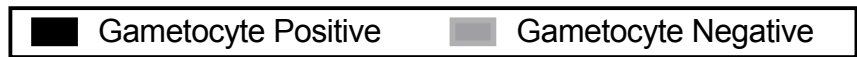

Supplement: Supplementary file 1 — Additional file 1. Age and gender effects on cross-sectional population gametocyte prevalence among the entire cohort during the wet and dry seasons. Gametocyte positivity was measured by RT-PCR once a month for 1 year. Data from November (A) and April (B) are presented as representative time-points for wet and dry seasons, respectively. Black bars and gray bars represent the proportion of gametocyte-positives and –negatives, respectively, within each category. Similar age and gender patterns were consistently observed at all other time-points throughout the year. [file 12936_2017_2123_MOESM1_ESM.pdf]
