# Supplementary material for: Host age and Plasmodium falciparum multiclonality are associated with gametocyte prevalence: a 1-year prospective cohort study
Source: Malar J. 2017 Nov 21;16:473. doi: 10.1186/s12936-017-2123-2 (PMC5696713; doi:10.1186/s12936-017-2123-2)
Supplement: Supplementary file 2 — Additional file 2. Effect of RBC polymorphisms on gametocyte positive rate. [file 12936_2017_2123_MOESM2_ESM.pdf]

Additional file 2: Effect of RBC polymorphisms on gametocyte positive rate

For each individual, gametocyte positivity throughout the study (number of gametocyte positive samples out of Pf positive samples) was calculated. Then multiple linear regression was performed using the gametocyte positivity as a determinant variable

Total Effect Test

	p-value
Age group	0.0003
Gender	0.4093
ABO	0.4288
Rh	0.9842
Hb Type	0.3161
G6PD deficiency	0.7626
α-thalassemia	0.1730

A total of 369 volunteers were Pf positive at least once in this study and included in the above analysis. The number of volunteers in each category is shown in the bottom tables

**Age group**	N
1-3 years	42
4-6 years	56
7-9 years	78
10-12 years	70
13-17 years	49
18-35 years	39
>35 years	35

**Gender**	N
Female	202
Male	167

**ABO**	N
A	119
B	98
AB	29
o	123

**Rh**	N
Positive	350
Negative	19

**Hb Type**	N
WT	284
AC	33
AS	49
CC	1
SC	2

**G6PD deficiency**	N
WT	308
A+/-	42
A-	19

**α-thalassemia**	N
WT	243
Hetero	118
Homo	8
