# Supplementary material for: Host age and Plasmodium falciparum multiclonality are associated with gametocyte prevalence: a 1-year prospective cohort study
Source: Malar J. 2017 Nov 21;16:473. doi: 10.1186/s12936-017-2123-2 (PMC5696713; doi:10.1186/s12936-017-2123-2)
Supplement: Supplementary file 3 — Additional file 3. Comparison of multiclonality between gametocyte-negative and -positive in Pf-positive individuals at four time-points. (A) Polymorphic proportion (PmP, proportion of polymorphic reactions among the total number of successful reactions) and (B) complexity of infection (COI, COIL-estimated number of unique Pf genotypes in each infection) among Pf-positive individuals at June (n = 119), November (n = 110), February (n = 51), and April (n = 42) time-points. For PmP, red boxes represent gametocyte-negative (Nega) and blue boxes represent gametocyte-positive (Posi) individuals. PmP and COI were compared between gametocyte carriers and non-carriers using Mann–Whitney and Chi square tests, respectively. Only the November time-point showed a significant difference in PmP (p = 0.029) or COI (p = 0.025) based on gametocyte status. [file 12936_2017_2123_MOESM3_ESM.pdf]

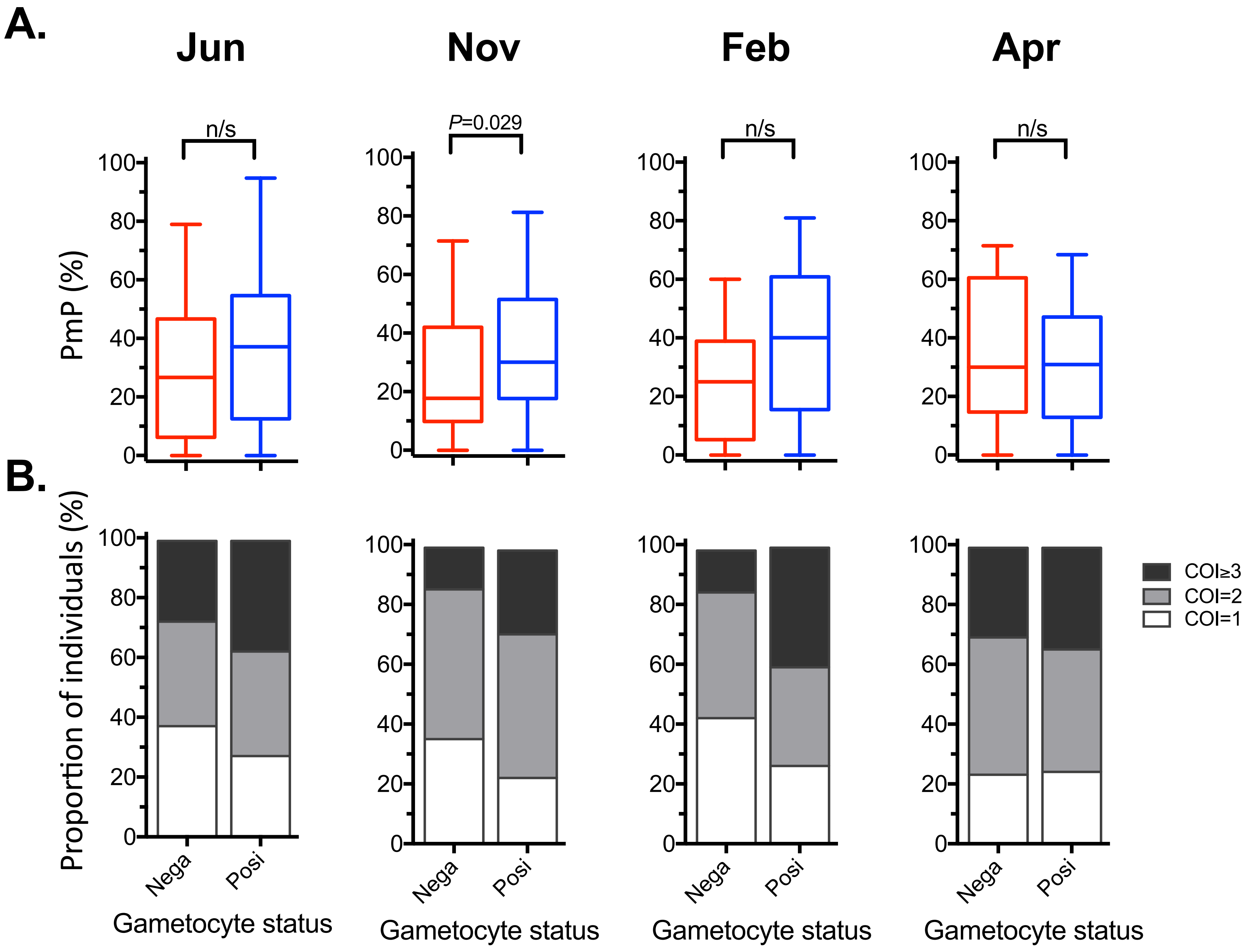

A.
Jun
Nov
Feb
Apr
n/s
P=0.029
n/s
n/s
PmP (%)
100
80
60
40
20
0
B.
Proportion of individuals (%)
COI≥3
COI=2
COI=1
Nega
Posi
Gametocyte status
